# Improving access to services in neuro-developmental disability: proceedings of a national meeting to advance community capacity

**DOI:** 10.1186/s12919-024-00311-3

**Published:** 2025-01-21

**Authors:** David B. Nicholas, Lucyna M. Lach, Samantha B. Sutherland, Joanne Maxwell, Stephanie McFarland, Anton R. Miller, Angela Clancy, Julie Scorah

**Affiliations:** 1 Department of Social Work, University of Calgary, Calgary, Canada; 2 School of Social Work, McGill University, Montreal, Canada; 3 Holland Bloorview Kids Rehabilitation Hospital, Toronto, Canada; 4 Department of Occupational Science and Occupational Therapy, University of Toronto, Toronto, Canada; 5 BC Children’s Hospital Research Institute, Vancouver, Canada; 6 Division of Developmental Pediatrics, Department of Pediatrics, University of British Columbia, Vancouver, Canada; 7 Family Support Institute of BC, Vancouver, Canada; 8 Azrieli Centre for Autism Research, Montreal Neurological Hospital-Institute, McGill University Health Centre, Montreal, Canada

**Keywords:** Neurodevelopmental disability, Children, Navigation, Service access, Capacity building

## Abstract

As part of a participatory project to advance navigational service delivery systems for children with neurodevelopmental disability (NDD) and their families, this paper addresses proceedings from a capacity-building conference in Vancouver, Canada. A total of 29 invited key stakeholders attended the meeting with the following aims: knowledge sharing amongst provincial/territorial regions advancing NDD navigation capacity; sustainable action-oriented knowledge exchange; and operationalizing next steps to build navigation resources across Canadian regions. Regional representation included multiple and inter-sectoral partners (e.g., not-for-profit organizations, government, education, health, researchers, etc.) strategically invited to address mutually-agreed upon regional challenges, where ideas for envisioning, planning and success could be developed for ultimate operationalization in three Canadian provinces and one territory based on need for building navigational service delivery systems in NDD.

Advancements in navigational service delivery were shared by site leads in the four represented regions of the initiative: Alberta, British Columbia, Quebec, and Yukon. Each regional lead conveyed targeted accomplishments, priorities and issues in moving navigation forward. Identified successes comprised the development of trusting partnerships across agencies and sectors, innovation and connection among service/navigation organizations and leaders, and training advances. Struggles included insufficient regional clarity on guiding principles for navigation services, a lack of resources relative to family need for services, and insufficient infrastructural supports in regions. Based on key learnings within and across regional groups, plans for regional development were strategized and shared.

## Introduction

Children with neuro-developmental disabilities (NDD) have a range of “congenital or acquired long-term conditions that are attributed to impairment of the brain and/or neuromuscular system and create functional limitations” (p. 1103) [1]. These children and their families typically require services to optimize development and integration in the community. Identified challenges include insufficient services relative to need, a lack of knowledge about services within regions and across sectors, rigid service eligibility criteria, prohibitive service costs, administrative processes such as lengthy application requirements and waitlists, services that are not coordinated, uncertainty about the quality of services, and a lack of knowledge about children’s rights to services [2]. Opportunities for improvements in service access include strategy development at a community level, with collaboration among service providers.

Addressing these areas for advancement includes, but is not limited to, reframing the nature of the problem, refocusing on systemic change strategies, and reimagining leadership [3]. The core concept of navigation has been applied among multiple populations and contexts, generally with the aim of reducing barriers to service access, connecting individuals and/or their families to the resources they need, integrating services in such a way that they are coordinated as opposed to siloed and disconnected, and providing information and emotional support to optimize individual and family well-being [4]. Timely and comprehensive service access in NDD is sought, requiring shifts to service delivery structures, more focus on continuity of care, system capacity/efficiency, and sustainability of innovation [5].

Despite these aims, primary health care providers, child and youth workers, social workers, teachers, lawyers, judges, as well as a host of other community service providers, typically struggle to address best approaches in efficiently finding services needed by children with NDD and their families. As a result of such system gaps, families too often experience significant strain as they independently try to navigate complex systems of care for their child. One study noted that with, “the lack of continuity of care between service sectors and over time, navigating systems of care was a ‘full-time job’ (for parents)” (p. 4) [6].

## Literature review

Navigation has become a widely used term, with reference to service wayfinding as well as the role of the navigator in providing support for service finding. Of 33 papers reviewed in a qualitative scoping review, 20 terms were found which pertained to navigation-type activities or services, while only 6 papers used the term: ‘navigation’ [7]. Recently, there has been a call for a more integrated approach to navigation via inter-agency partnership and training for parents and service providers in gaining a better understanding of how to ensure families find the services they need *when* they need them.

Such navigation-like activities and/or programs are viewed to respond to insufficient services or a confusing array of services in the system. It is proposed that if a clearer scope of services and navigational support was offered, notable benefits for families would result [8]. To redress the current reality of insufficient navigational support in finding services, stakeholders from four Canadian regions (Alberta, British Columbia, Quebec, Yukon) have worked to develop a navigation-building framework to facilitate help-seeking and ensure improved access to these supports. Objectives of this four-year system-shifting initiative were to understand gaps and strategize and implement capacity-building to ensure system-level navigation advancement [2]. To date, the work of each respective region entailed partnership with community-based service providers, government representatives, family advocates, and advocacy organizations.

A strategy-planning meeting was held to address these issues and share strategies for advancement. The specific aims of the meeting were to generate learning from regional initiatives relative to challenges, strategies, successes and lessons learned in advancing capacity. Capacity building is described as, “incremental in phases, but nonlinear and dynamic; the change process takes time; development is essentially about developing people; development builds on existing resources; development cannot be imposed from the outside; and development is ongoing” (p. 143) [9]. Capacity building was sought by identifying potential steps for moving forward [10]. A total of 29 people attended the strategy planning meeting comprising key service navigation leaders/representatives from three Canadian provinces and one territory, as listed above. Input from each province and territory was prioritized to gain a deeper understanding of what is needed regionally and from diverse points of view. Below is an outline of meeting content and key learning/reflection of the delegates.

## Navigation meeting proceedings (Methods)

The Navigation meeting opened with an introduction and welcome from Dr. David Nicholas, Professor and Associate Dean of Research and Partnerships, Faculty of Social Work, University of Calgary and Dr. Lucyna Lach, Associate Professor, School of Social Work, Faculty of Arts, McGill University. Drs. Nicholas and Lach outlined current gaps in the service delivery system, as outlined above, and the need for sufficient navigation support relative to the needs of families, both individually relative to child/family needs and as an integrated network of services in regions. Examples of innovative mitigative strategies were introduced, as follows.

## Collective learning and knowledge sharing

Angela Clancy, Executive Director of the Family Support Institute of British Columbia, described the development of a professional capacity-building portal for navigators in NDD. The portal will offer navigation support and training for navigators who are supporting individuals with disability and their families, and will include resources across sectors which is anticipated to advance access to navigation information resources and training for service providers. The collaborative design process prioritizes the provision of feedback from professionals to respond to emerging needs with up-to-date information. Innovations include a “spider” set-up on the website, consisting of a program running every two weeks to flag program/website changes for review by an administrator, thus continually updating content. The portal offers sustained learning for service providers including updated resources, navigator training and professional development.

## Navigation approaches to support social needs and transitions to adulthood

Stephanie McFarland and Joanne Maxwell, Occupational Therapist & Manager and Senior Director, respectively, at the Family Navigation Hub, Holland Bloorview Kids Rehabilitation Hospital in Toronto, presented a novel navigation resource to support family access to services, with a focus on redressing barriers related to social determinants of health in marginalized communities. Areas of inquiry relative to resource sufficiency are: food security, housing, coverage of utilities, childcare, health care cost coverage, transportation, health literacy, and lastly, social and community connection. A *Social Needs Screening Tool* is administered to ascertain barriers which could impede well-being and/or access to care as well as identified levels of urgency. This screening tool was trialed in six programs (n = 165) with 59% of families indicating at least one area of social need for which they sought support, and 70% of families identified needs as urgent. Future aims of this team include building organizational capacity to determine effectiveness in addressing needs. Discussion among delegates addressed the need to proactively identify and address equity-based barriers to service access, and this approach serves as an example of targeting these needs.

## Developing training and capacity: Extension for Community Healthcare Outcomes (ECHO)

Training and support for service providers is an emerging gap and priority in moving forward. A presentation by Dr. Julie Scorah, Neuropsychologist, and Assistant Professor, Department of Neurology and Neurosurgery, McGill University, addressed the work and training foundation of ECHO Autism. ECHO Autism’s mission is to ensure broad access to expertise and disseminate best practices, mentor and guide more remote communities of clinicians, educators and advocates, thus creating local expertise and increasing access to services for all autistic individuals and their families.

Principles of the ECHO Model are to utilize technology in leveraging scarce resources, share best practices, offer case-based learning to master complexity, and monitor outcomes. Ultimately, these elements aim to contribute to a learning community in which “all teach, and all learn.” Results from a pilot trial in ten sites by researchers in the United States [11] were reported to have demonstrated an increase in overall self-efficacy in NDD screening, diagnosis, and management. Noted impacts were further reported to include: mentorship for a range of relevant specialties, expansion of local expertise and best practices, individual practitioner skill development, and holistic care to individuals and families near their home.

Regional representatives strongly recognized the value of the ECHO model, and considered training needs and priorities across regions, with a proposed plan to advance national training and capacity development via a future national grant. Such an endeavor is needed and promising in advancing training and evaluation, and ensuring that navigational resources are broadly available even in rural and remote regions.

## National and regional navigation capacity-building

Drs. Nicholas and Lach presented lessons learned from capacity building across the four target regions of this initiative. In seeking capacity building, they reflected on the importance of clarity of purpose, team development, and partnership building, with this learning galvanized in the recently published “Community Guide – Navigation: Building Community Capacity in Neurodisability Services.” Building on outcomes of this project, this Guide offers practical steps to increase capacity for improved family access to health and social care-related programs and services, barrier reduction, and efficient use of health and social care systems. Sections of the Guide include processes to enhance navigation services, including building community connection, setting the stage for change, capacity advancement, and sustainability of change. The following specific capacity building goals are addressed: (1) share key insights about how to build navigational capacity in a community or jurisdiction, (2) delineate key processes for team and initiative development, (3) indicate core markers of evaluation, and (4) outline strategies to optimize sustainability. To obtain the Community Guide, go to the *Building Community Capacity for Neurodivergent Individuals* website (www.bccndi.org).

## Regional Advancement of Navigation Services (Results)

The four regional site leads of the Project shared updates on the advancement of navigation services.. Presentations reflected advances in each region including reflections on key issues such as partnership-building, training, strategy/re-imagining, and plans for sustainability. Summary of this work for each region is outlined below.

### Alberta

Dr. Nicholas spoke about advancing navigation services in Edmonton and surrounding regions. Team members include a steering committee with representation by family members/advocates, service providing organizations and government. This initiative has determined how various organizations are contributing to navigation services in the region, with an aim of heightening awareness, capacity and clarity of each other’s roles, and determining needs in moving forward. Emerging opportunities for system capacity building have been highlighted in the development and proposed implementation of an integrated provincial approach to NDD navigation.

**Fig. 1:**
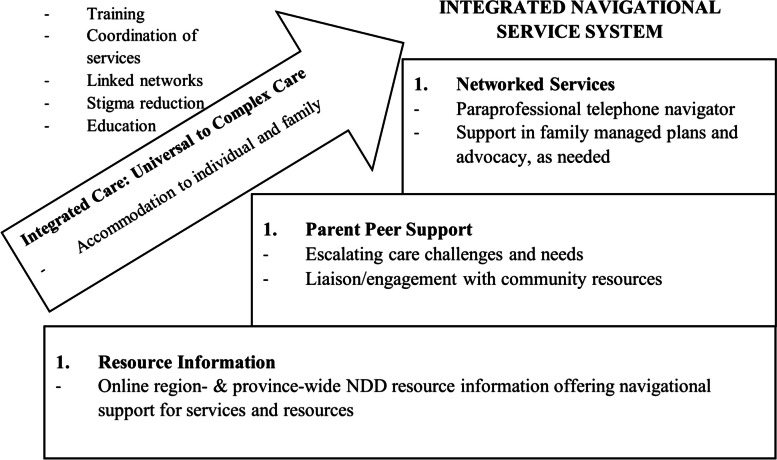
Integrated navigational service system

A province-wide model is being sought in advancing a navigation support system that leverages partnerships that collectively reduce service duplication. Summarized in Fig. 1, this navigation approach entails accessible online resource information, parent-to-parent peer support, and personalized navigational counselling and advocacy tailored to family needs (information and referrals). The approach is noted to offer a continuum of supports from universal to complex, based on family need.

The Alberta team is rolling out this integrated NDD navigation network among a consortium of service providing partners. The team further is developing online navigation training modules, in partnership with the Northern Alberta Institute of Technology, University of Calgary, University of Alberta, Glenrose Rehabilitation Hospital, Alberta 211, and Alberta Caregiver College, for distribution to navigators (see training modules at https://caregivercollege.ca/course/index.php?categoryid=5_). Within this initiative, they are researching how to optimally recruit, orient, and support parent/peer mentors in offering navigational support at a provincial level.

### British Columbia (BC)

Leaders of the BC project, Angela Clancy of the Family Support Institute of BC, and Dr. Anton Miller of the Sunny Hill Health Centre and the University of British Columbia, shared regional progress which included the establishment of a Provincial Advisory Group (PAG), identifying and addressing key barriers to navigation advancement, namely the lack of a shared understanding about what comprises navigation and the need for more clarity about one’s own and other agencies’ roles and scopes in navigation. These areas for developing clarity were addressed through exploratory research and dialogue among navigation-based agencies who sought greater specificity and a shared understanding regarding the terminology and work of navigation. Two regional summits in BC were held in making connection across key organizations and collectively sharing knowledge (https://www.bcacdi.org/navigation-project) [4]. A reported need from this work was to amplify family support and navigation support; accordingly, this regional team has worked to advance avenues of communication, collaboration, and engagement.

Along with a doctoral candidate Jeff McCrossin at McGill University, FSI-BC evaluated families’ experiences of navigation and family support. Based on evaluation findings, while the complexities and challenges experienced by families were similar, the type of support families received varied. This highlights the importance of support options that are tailored to the needs of each family [12].

The team identified that families report the need for early access and follow-up through a navigator network. Elements of such a network entail support, connection, enhanced professional training, and increased individual and family capacity. Comprehensiveness and inclusivity of resources were reported to be needed, however, factors such as high demand for service providers, limited capacity within the system, and budget pressures need to be addressed in building system capacity. Discussants emphasized the importance of addressing pressing considerations in an integrative way that addresses barriers while also honoring local contexts.

### Quebec

Dr. Lach spoke about the regional aim and priority of supporting a life of quality for adults with NDD – the focus of capacity building by the team from Montreal, Quebec. Specifically, the Rehabilitation Advisory Committee of the CIUSSS Centre-Ouest has advanced the aim of generating policy briefs pertaining to continuing education and employment, housing, governance, and services that promote physical, emotional and social well-being of neurodivergent adults and their families. Directed at the regional health authority and to the provincial government, each generated recommendation would strengthen existing service offerings. The partnerships developed between sectors and community organizations have contributed to the co-creation and visibility of pathways for accessing support and services that are both needed and evidence-based. The team is focusing on individuals with NDD transitioning from adolescence to adulthood.

In moving this work forward, the Quebec team identified a priority of addressing what has been a largely siloed system of organizations, and to rather seek integration in support provision. This work has guided this team to recommend a ‘table’ or inter-ministerial Secretariat where concerns and priorities can be holistically addressed. To that end, a collaborative structure and associated processes of support would be identified and advanced.

### Yukon

Wenda Bradley, Project Lead Yukon and former Executive Director, Fetal Alcohol Syndrome Society Yukon (FASSY), presented NDD navigation advancements in the Yukon. A key advancement was the establishment of a community interagency committee in 2017, with goals of creating greater community capacity and increased support for people with NDD within existing services. Emerging successes were identified as strategy development for redressing service access gaps, as well as seeking increased employment and community knowledge about the needs of individuals with NDD and their families.

In moving forward, ingenuity in working proactively relative to Indigenous ways of knowing, being and doing have been prioritized. This includes programming that is respectful of Indigenous culture such as collaboration with community partners to offer land-based services for individuals with FASD and their families. Such innovation and areas of program development were noted as integral in responding to Truth and Reconciliation commitments through engagement and healing supports offered in culturally-sensitive ways. Developing and sustaining strong relationships were noted as key to programmatic success.

### Regional steps for advancement and impact

In moving forward, the delegation identified the need for greater clarity of guiding principles for advancing navigational supports in Canadian jurisdictions. The following principles guiding capacity development were proposed: person and family-centeredness, flexibility relative to community need, collaborative and cooperative practice, relationality, trust-based partnership, accessibility, transparency in communication, cultural relevance, and trauma-informed practice.

A lingering and pressing concern to address across jurisdictions was insufficient resources relative to service need for individuals and families, and human resource shortages in this sector. Advocacy was identified as critical via continued and increased partnership, engaged leadership, and collective capacity building. To advance such collective learning and pressing priorities, regional representatives advocated continued engagement to promote navigation development, sector identity development, peer support and mentorship in nurturing community and capacity.

Advocacy for more resources, collectively building knowledge and inspiring human resource recruitment were addressed. The delegation conveyed a desire for, and commitment to training development for navigators. Priorities included practice development in the field; as an example, delegates spoke about navigational interventions whereby navigational support is tailored to where an individual/family is at.

## Discussion

Regions across Canada (or other world regions) bring jurisdictional needs for advancing NDD navigational support capacity. To that end, the collective work of the represented regions is moving forward, both individually as regions and collectively at a national level, including mutual learning reflective of sharing gains and lessons within and across jurisdictions. Evaluation of outcomes and knowledge/innovation sharing is needed.

To address system-based gaps, steps, “are incremental and sometimes overlapping [within] a broad community change effort at multiple levels, from micro (individual capacity building) to mezzo (organizational capacity building), and macro (societal change)” (p. 109) [13]. Partners in this initiative have worked to understand navigation in terms of their regional priorities, along with action plans. There is critical need to integrate key collective impact elements: (i) a common agenda, (ii) shared measurement, (iii) mutually reinforcing activities, (iv) continuous communication, and (v) backbone support from the university/research and community project-steering teams [14]. This will require continued attention to address questions such as, ‘what is our collective agenda?’, ‘how are we measuring what we are doing?’, ‘what mutual engagement exists?’, ‘how are we going to best communicate key messages?’, ‘how are we going to accomplish a developmental evaluation?’, and ‘how do we support sustained change regionally and nationally?’.

The many connections and partnerships within regions and across the country from this initiative have resulted in capacity building that has contributed to jurisdictional advancement. In this advancement, a central question has guided, and continues to guide this work, “How can we improve navigation service access and quality of life for individuals with NDD and their families?" Such pivotal inquiry propels this continuing journey of community capacity building and has compelled important work forward in advancing navigation in NDD.
